# Semaglutide for Refractory Reactive Hypoglycaemia: A Case Report

**DOI:** 10.7759/cureus.112861

**Published:** 2026-07-17

**Authors:** Harshni Kalimuthu, Gayathma Seekkuge, Sona Joseph

**Affiliations:** 1 Critical Care Medicine, Christian Medical College, Vellore, IND; 2 Diabetes and Endocrinology, Doncaster Royal Infirmary, Doncaster, GBR

**Keywords:** continuous glucose monitoring, glp-1 receptor agonist, postprandial hypoglycaemia, reactive hypoglycaemia, semaglutide

## Abstract

Reactive hypoglycaemia is characterised by symptomatic hypoglycaemia occurring several hours after food intake and can significantly impair quality of life. Management is often challenging, particularly in patients who fail dietary modification or cannot tolerate conventional pharmacological therapies. We present the case of a 50-year-old woman with longstanding, debilitating reactive hypoglycaemia who experienced marked symptomatic and biochemical improvement following treatment with semaglutide, a glucagon-like peptide-1 (GLP-1) receptor agonist.

The patient presented with recurrent episodes of dizziness, profound fatigue, tremulousness, and syncope despite adherence to dietary recommendations. Previous treatment with acarbose was discontinued because of gastrointestinal intolerance, and metformin failed to improve symptoms. Continuous glucose monitoring and an oral glucose tolerance test confirmed reactive hypoglycaemia. Following initiation of semaglutide, the patient experienced a substantial reduction in hypoglycaemic episodes with corresponding improvement in glucose profiles and quality of life.

This case highlights the potential role of semaglutide as a novel therapeutic option in patients with refractory reactive hypoglycaemia and contributes to the emerging evidence supporting the use of GLP-1 receptor agonists in this condition.

## Introduction

Reactive hypoglycaemia, also known as postprandial hypoglycaemia, is characterised by symptomatic hypoglycaemia occurring within several hours after food ingestion and may significantly impair daily functioning and quality of life [1,2]. In susceptible individuals, ingestion of carbohydrate-rich meals may produce a rapid rise in blood glucose, provoking an exaggerated insulin response that subsequently causes glucose concentrations to fall. This results in adrenergic symptoms, such as tremulousness and palpitations, together with neuroglycopenic manifestations, including dizziness, fatigue, confusion, and, in severe cases, syncope [1,2]. Although dietary modification remains the cornerstone of management, the condition is considered refractory when clinically significant symptoms persist despite appropriate nutritional intervention and conventional pharmacological therapy [2].

The pathophysiology of reactive hypoglycaemia is complex and incompletely understood. Proposed mechanisms include exaggerated or delayed postprandial insulin secretion, altered incretin physiology, rapid gastric emptying, increased insulin sensitivity, and impaired counter-regulatory hormonal responses. Following an initial postprandial rise in glucose, insulin secretion may remain excessive relative to the declining glucose concentration, resulting in late postprandial hypoglycaemia. This mechanism explains the apparently paradoxical occurrence of low blood glucose after food intake.

Management primarily involves dietary modification, including frequent small meals, avoidance of refined and high-glycaemic-index carbohydrates, and increased intake of protein and complex carbohydrates to minimise rapid postprandial glucose fluctuations [2]. Pharmacological therapies, such as acarbose and metformin, have also been used with variable success; however, their effectiveness is inconsistent and gastrointestinal adverse effects frequently limit long-term adherence [2].

Glucagon-like peptide-1 (GLP-1) receptor agonists, including semaglutide, are established therapies for type 2 diabetes mellitus and obesity [3-6]. Their use in patients with recurrent hypoglycaemia may initially appear paradoxical because these medications are widely recognised for lowering blood glucose. However, emerging evidence suggests that GLP-1 receptor agonists may improve postprandial glycaemic stability by slowing gastric emptying, attenuating rapid postprandial glucose excursions, reducing exaggerated insulin responses, and modulating glucose-dependent glucagon secretion [3-6]. By blunting the initial postprandial glucose peak, these agents may reduce the subsequent insulin overreaction responsible for late postprandial hypoglycaemia. Although GLP-1 receptor agonists have a relatively low intrinsic risk of hypoglycaemia because their insulinotropic effects are glucose-dependent, post-marketing surveillance has reported rare episodes of hypoglycaemia, and their use in reactive hypoglycaemia remains off-label, warranting careful patient selection and monitoring [6].

Most published evidence supporting GLP-1 receptor agonists in postprandial hypoglycaemia has been derived from case reports, case series, and studies involving patients following bariatric surgery [7]. Evidence supporting their use in non-bariatric refractory reactive hypoglycaemia remains limited, and the optimal agent, dose, treatment duration, long-term safety, and patient selection criteria have yet to be established.

We report the case of a patient with longstanding non-bariatric refractory reactive hypoglycaemia who remained symptomatic despite dietary modification and conventional pharmacological therapy. Clinical improvement and favourable continuous glucose monitoring findings were observed following initiation of semaglutide. Given the observational nature of this report, these findings represent a temporal association rather than evidence of causality and contribute to the emerging evidence supporting further investigation of GLP-1 receptor agonists in carefully selected patients with refractory reactive hypoglycaemia.

## Case presentation

A 50-year-old woman with a body mass index (BMI) of approximately 30 kg/m² and a baseline weight of 70 kg presented to the endocrinology clinic with a 10-year history of recurrent postprandial hypoglycaemic episodes, which had progressively worsened over the preceding 2 years. She described episodes occurring consistently two to four hours after meals, characterised by tremulousness, palpitations, dizziness, profound fatigue, and occasional syncope. The increasing frequency and severity of these episodes had significantly impaired her ability to work, perform daily activities, and participate in social engagements.

The episodes occurred predominantly after meals rich in refined or high-glycaemic-index carbohydrates. Despite adherence to dietary recommendations, including frequent small meals, avoidance of refined carbohydrates, increased protein intake, and consumption of low-glycaemic-index foods, she continued to experience approximately six symptomatic hypoglycaemic episodes per week. The patient had no history of bariatric surgery, gastric surgery, or other gastrointestinal procedures.

Previous pharmacological therapy included acarbose 50 mg three times daily, which was discontinued because of severe gastritis and abdominal discomfort, followed by a trial of metformin 500 mg twice daily, which failed to produce meaningful symptomatic improvement. As symptoms persisted despite appropriate dietary modification and conventional pharmacological therapy, the condition was considered refractory reactive hypoglycaemia.

Baseline laboratory investigations are summarised in Table 1. HbA1c was 38 mmol/mol (5.6%), with normal fasting plasma glucose, fasting insulin, C-peptide, thyroid function, morning cortisol, renal function, and liver function tests.

**Table 1:** Baseline biochemical investigations Abbreviations: HbA1c, glycated haemoglobin; TSH, thyroid-stimulating hormone Reference ranges are those used by our institution's laboratory.

Investigation	Result	Reference Range
HbA1c	38 mmol/mol(5.6%)	20-41 mmol/mol
Fasting plasma glucose	4.8 mmol/L	3.9-5.5 mmol/L
Fasting insulin	8.4 mIU/L	2-25 mIU/L
C-peptide	2.1 ng/mL	0.9-4.0 ng/mL
Morning cortisol	468 nmol/L	140-690 nmol/L
TSH	2.1 mIU/L	0.4-4.0 mIU/L
Renal function	Within normal laboratory limits	-
Liver function tests	Within normal laboratory limits	-

Oral glucose tolerance testing (OGTT) demonstrated a characteristic postprandial glucose excursion followed by late hypoglycaemia, with plasma glucose decreasing to 3.2 mmol/L at 180 minutes (Table 2). The diagnosis was based on the characteristic clinical presentation together with documented biochemical hypoglycaemia occurring during the postprandial period. Although symptom resolution following glucose administration was not formally documented, the overall findings were considered consistent with reactive hypoglycaemia.

**Table 2:** Oral glucose tolerance test results Reference range: Fasting plasma glucose: 3.9-5.5 mmol/L. Hypoglycaemia is generally defined as plasma glucose <3.9 mmol/L.

Time	Plasma Glucose (mmol/L)
Fasting	4.8
30 minutes	8.9
60 minutes	10.4
90 minutes	8.1
120 minutes	5.2
180 minutes	3.2

Alternative causes of recurrent hypoglycaemia were considered. The absence of fasting hypoglycaemia, normal fasting insulin and C-peptide concentrations, normal endocrine investigations, and the consistent postprandial pattern of symptoms supported a diagnosis of reactive hypoglycaemia rather than fasting hyperinsulinaemic hypoglycaemia. A supervised 72-hour fasting test, pancreatic imaging, insulin and C-peptide measurements during the hypoglycaemic phase of the OGTT, and screening for autoimmune hypoglycaemia were not performed during routine clinical evaluation. While these investigations may assist in excluding alternative causes such as insulinoma in selected patients, their absence is acknowledged as a limitation of this report.

Continuous glucose monitoring (CGM) was performed using the FreeStyle Libre system (Abbott Laboratories, Abbott Park, IL, Noida) before treatment and repeated at the six-month follow-up. Baseline CGM demonstrated recurrent postprandial hypoglycaemia, with a lowest recorded glucose concentration of 3.2 mmol/L, approximately 6 symptomatic hypoglycaemic episodes per week, and 12% time below range (TBR; <3.9 mmol/L).

Given persistent symptoms despite lifestyle modification and previous pharmacological therapy, semaglutide was initiated at 0.25 mg once weekly and increased to 0.5 mg once weekly after four weeks. The patient reported a gradual improvement in symptom frequency during dose escalation, with further reduction in symptom severity after reaching the maintenance dose. By six weeks, she reported no further syncopal episodes and a marked improvement in energy levels and confidence in performing daily activities.

Repeat CGM obtained at the six-month follow-up demonstrated a reduction in hypoglycaemic burden, with symptomatic hypoglycaemic episodes decreasing from six to one episodes per week and time below range improving from 12% to 1% (Table 3). Because this report describes the clinical course of a single patient, formal statistical analysis was not appropriate and was therefore not performed.

**Table 3:** Continuous glucose monitoring findings Reference range: Lowest glucose (mmol/L) - 3.9-7.8 *CGM = Continuous Glucose Monitoring; TBR = Time Below Range A symptomatic hypoglycaemic episode was defined as a distinct clinical event associated with sensor glucose <3.9 mmol/L.

Parameter	Before Semaglutide	After Semaglutide
Lowest glucose recorded	3.2 mmol/L	4.0 mmol/L
Hypoglycemic episodes/week*	6	1
Time below range (TBR) (<3.9 mmol/L)	12%	1%

The patient continued semaglutide without interruption, and the improvement was maintained throughout the six-month follow-up. During this period, her weight decreased from 70 kg to 58 kg. Although weight loss may have contributed to improved metabolic control, the relative contribution of weight reduction compared with the direct pharmacological effects of semaglutide cannot be determined from this single case. The patient also reported subjective improvement in daily functioning and reduced anxiety related to recurrent hypoglycaemic episodes. As validated quality-of-life instruments were not used, these observations are based on patient-reported outcomes. Gastrointestinal adverse effects were specifically monitored because of previous intolerance to acarbose; however, no clinically significant nausea, vomiting, abdominal pain, worsening hypoglycaemia, or treatment discontinuation due to adverse effects was observed.

## Discussion

Reactive hypoglycaemia is a challenging clinical condition characterised by recurrent symptomatic hypoglycaemia occurring several hours after food ingestion. While dietary modification remains the cornerstone of management, some patients continue to experience debilitating symptoms despite lifestyle interventions and conventional pharmacological therapy. In such cases, therapeutic options are limited, and evidence guiding treatment is sparse. In this report, a patient with longstanding refractory reactive hypoglycaemia experienced sustained clinical improvement following semaglutide therapy after unsuccessful treatment with dietary modification, acarbose, and metformin. Although causality cannot be established from a single case report, the temporal association between semaglutide initiation and improvement in both symptoms and CGM findings suggests a potential therapeutic role that merits further investigation [1,2].

At first glance, the use of a GLP-1 receptor agonist to treat recurrent hypoglycaemia appears paradoxical, as semaglutide is primarily recognised as a glucose-lowering therapy for type 2 diabetes mellitus and obesity. However, GLP-1 receptor agonists exert multiple physiological effects beyond reducing blood glucose. By slowing gastric emptying, they delay intestinal glucose absorption and attenuate the magnitude of the initial postprandial glucose excursion. Consequently, the exaggerated insulin response that precipitates reactive hypoglycaemia may also be reduced. Furthermore, GLP-1-mediated insulin secretion is glucose dependent, meaning insulin release diminishes as plasma glucose falls, contributing to the low intrinsic risk of hypoglycaemia when these agents are used without insulin or sulfonylureas. These mechanisms provide a biologically plausible explanation for the clinical improvement observed in our patient [3-5].

In addition to their effects on gastric emptying and insulin secretion, GLP-1 receptor agonists influence glucagon physiology. Glucagon secretion is suppressed during hyperglycaemia, but this effect diminishes as plasma glucose approaches the normal range, thereby preserving physiological counter-regulatory responses during hypoglycaemia. Although the exact contribution of glucagon modulation to reactive hypoglycaemia remains uncertain, this glucose-dependent mechanism may further support glycaemic stability without substantially increasing the risk of severe hypoglycaemia [7].

Semaglutide was selected for this patient because symptoms persisted despite first-line dietary measures and conventional pharmacological therapy. Acarbose is frequently recommended for reactive hypoglycaemia because it delays carbohydrate absorption; however, gastrointestinal adverse effects often limit long-term adherence, as occurred in our patient. Metformin has also been reported to improve reactive hypoglycaemia in selected individuals, possibly by reducing hepatic glucose production and improving insulin sensitivity, but symptomatic benefit is inconsistent. More recently, Younes et al. proposed a treatment strategy incorporating metformin and GLP-1 receptor agonists for patients with refractory reactive hypoglycaemia, reflecting increasing interest in targeting postprandial glucose excursions rather than hypoglycaemia alone. Compared with shorter-acting GLP-1 receptor agonists such as liraglutide, semaglutide offers once-weekly administration, sustained receptor activation, and excellent treatment adherence. However, comparative studies evaluating different GLP-1 receptor agonists in reactive hypoglycaemia are lacking, and no agent can currently be recommended over another based on available evidence [7-9].

The published literature evaluating GLP-1 receptor agonists in reactive hypoglycaemia remains limited. Most available evidence relates to post-bariatric hypoglycaemia, where exaggerated endogenous GLP-1 secretion contributes to excessive postprandial insulin release. Abrahamsson et al. first reported symptomatic improvement with liraglutide in patients with postprandial hyperinsulinaemic hypoglycaemia following gastric bypass surgery, suggesting that modulation of postprandial glucose excursions may reduce subsequent hypoglycaemia rather than exacerbate it [9]. More recently, a systematic review concluded that GLP-1 receptor agonists may reduce hypoglycaemic episodes in selected patients with post-bariatric hypoglycaemia, although the available evidence remains limited by small sample sizes and a lack of randomised controlled trials. Similarly, Fiore et al. described the successful use of semaglutide in a patient with dumping syndrome-related reactive hypoglycaemia after bariatric surgery [10]. In contrast, evidence supporting the use of GLP-1 receptor agonists in non-bariatric reactive hypoglycaemia is scarce. Consequently, our case adds to the emerging literature suggesting that semaglutide may have a therapeutic role beyond the post-bariatric population and highlights the need for prospective studies in this under-recognised group.

An important strength of this case is the availability of objective CGM data before and after treatment. Following semaglutide therapy, symptomatic hypoglycaemic episodes decreased from six to one episode per week, while time below range improved from 12% to 1%, supporting a clinically meaningful reduction in hypoglycaemic burden. We intentionally avoid concluding that semaglutide reduced glycaemic variability, as standard CGM-derived measures, such as coefficient of variation, glucose standard deviation, ambulatory glucose profile, and time in range, were not available. Likewise, although the patient reported improved energy levels, confidence in daily activities, and overall well-being, validated quality-of-life instruments were not used. These observations, therefore, represent subjective patient-reported outcomes rather than objectively measured clinical endpoints [11-13].

During follow-up, the patient also experienced weight loss. Although this may have contributed to improved metabolic regulation and insulin sensitivity, the relative contribution of weight reduction compared with the direct physiological effects of semaglutide cannot be determined from a single case. Consequently, it remains uncertain whether the observed improvement resulted primarily from attenuation of postprandial glucose excursions, weight loss, or a combination of both mechanisms. Future studies incorporating detailed metabolic assessment may help clarify the relative importance of these factors [14-17].

Limitations

Several limitations should be acknowledged. As a single-patient case report, this study cannot establish causality or determine treatment efficacy. Formal statistical analysis, including p-values or confidence intervals, was neither appropriate nor possible. A control period, washout phase, or treatment rechallenge was not performed, limiting causal inference. Although fasting insulin and C-peptide concentrations were normal, insulin and C-peptide were not measured during the hypoglycaemic phase of the oral glucose tolerance test, preventing confirmation of exaggerated postprandial hyperinsulinaemia. Similarly, a supervised 72-hour fasting test, pancreatic imaging, and investigations for autoimmune hypoglycaemia were not undertaken. While the patient's characteristic postprandial symptoms, documented biochemical hypoglycaemia, and absence of fasting hypoglycaemia strongly supported a diagnosis of reactive hypoglycaemia, these additional investigations may have further excluded alternative causes such as insulinoma. Furthermore, comprehensive CGM metrics, including time in range, glucose variability, and ambulatory glucose profile, were unavailable, and validated quality-of-life questionnaires and structured dietary adherence assessments were not performed. These limitations should be considered when interpreting the findings.

Despite these limitations, this case has important clinical implications. Patients with refractory reactive hypoglycaemia often experience considerable impairment in quality of life despite adherence to recommended dietary strategies and available pharmacological therapies. Our findings suggest that semaglutide may represent a promising therapeutic option in carefully selected patients who remain symptomatic despite conventional management. However, this observation should be regarded as hypothesis-generating rather than practice-changing. Larger prospective studies incorporating comprehensive metabolic phenotyping, standardised CGM reporting, validated patient-reported outcome measures, and longer follow-up are required to clarify the mechanisms underlying the observed response, identify patients most likely to benefit, and establish the efficacy and safety of GLP-1 receptor agonists in refractory reactive hypoglycaemia.

## Conclusions

This case demonstrates the potential role of semaglutide as a therapeutic option for refractory reactive hypoglycaemia in a patient who remained symptomatic despite dietary modification, acarbose, and metformin. Treatment was temporally associated with sustained clinical improvement, including a marked reduction in symptomatic hypoglycaemic episodes and objective improvement in continuous glucose monitoring parameters. The observed benefit is biologically plausible given the effects of GLP-1 receptor agonists on gastric emptying, postprandial glucose excursions, and glucose-dependent insulin secretion. However, as this is a single case report, causality cannot be established, and the contribution of factors, such as weight loss, cannot be excluded. This report should therefore be regarded as hypothesis-generating rather than practice-changing. Larger prospective studies incorporating comprehensive metabolic assessment, standardised continuous glucose monitoring metrics, and validated patient-reported outcome measures are required to determine the efficacy, safety, and optimal role of GLP-1 receptor agonists in the management of refractory reactive hypoglycaemia.
